# Adolescents’ Sexy Self-Presentation on Instagram: An Investigation of Their Posting Behavior Using a Prototype Willingness Model Perspective

**DOI:** 10.3390/ijerph17218106

**Published:** 2020-11-03

**Authors:** Joris Van Ouytsel, Michel Walrave, Mónica Ojeda, Rosario Del Rey, Koen Ponnet

**Affiliations:** 1 Department of Communication Studies, MIOS, University of Antwerp, 2000 Antwerp, Belgium; michel.walrave@uantwerpen.be; 2 Department of Educational and Developmental Psychology, Universidad de Sevilla, 41013 Sevilla, Spain; monicaojeda@us.es (M.O.); delrey@us.es (R.D.R.); 3 Department of Communication Studies, IMEC-MICT, Ghent University, 9000 Ghent, Belgium; Koen.Ponnet@UGent.be

**Keywords:** sexting, sexualized self-presentation, social media, e-safety

## Abstract

Some adolescents use social media platforms, such as Instagram, for sexualized self-presentation, which includes posting images in which someone is scarcely dressed, has a sexy gaze or in which sexual willingness is suggested. These behaviors could be a first step towards sexting. Given that adolescents are highly influenced by peer perceptions, this study uses the prototype willingness model to assess how teenagers’ perceptions of others could influence their posting behaviors. The study was conducted among 2626 students (*n* = 1530; 58.4% girls) between the ages of 14 and 21 (*M* = 16.14; *SD* = 1.02) in 10 secondary schools in the Dutch-speaking community in Belgium. The results show that older adolescents and girls were more likely to post images of themselves on Instagram. The models showed that peer norms and willingness and attitudes were significantly associated with posting intention. The perceived norms of adolescents’ parents were not significantly related to the behaviors. There were also significant associations between perceived similarity, prototype favorability and the willingness to engage in sexualized self-presentation on Instagram. The implications for education and practice are discussed.

## 1. Introduction

Adolescents are enthusiastic social media users. Around 95% of US teenagers own a smartphone and 45% are almost permanently online [1]. Additionally, in Europe, smartphones have become the preferred medium for online communication. Most teenagers use them daily or almost all the time [2]. One of the social media applications that has gained a lot of popularity over the recent years is Instagram. In the United States, 72% of youth are active users of the platform. In Belgium, where the current study was conducted, around 86% of youth report using Instagram on a weekly basis in 2019 [3]. Instagram is a social networking application that allows users to share images and videos of themselves. The content can either be permanently posted on the timeline or can be published as temporary posts that stay up for a limited amount of time (i.e., Instagram stories). Instagram solicits feedback from others, by allowing users to like the content and post comments under images, which can be perceived by its users as measures of success and validation [4].

Similar to other social networking platforms, Instagram is used by adolescents for social interaction, such as staying in touch with friends and making new connections [5]. From a developmental perspective, social media platforms allow teenagers to experiment with their identities, online personas, and their looks, as they allow receiving feedback from others through comments and likes [6,7]. One study has found that validation and reassurance seeking are one of the main motives for why adolescents use Instagram. It was found that teenagers use the social media platform in order to improve their peer popularity and to express their creativity [5]. In this study, we focus on how teenagers can use the social media platform to engage in sexual forms of self-presentation. In order to get a deeper understanding of adolescents’ engagement in sexualized self-presentation on Instagram, our study uses the prototype willingness model (PWM) [8].

## 2. Literature Review

Given their focus on visual content, social media applications such as Instagram have been named as platforms that adolescents can use for sexualized self-presentation, which, based on the conceptualization of van Oosten [9], includes images in which someone is scarcely dressed, has a sexy gaze, or in which sexual willingness is suggested. Engaging in sexualized self-presentation appears to be common among adolescents. A content-analysis of profile pictures on a chat platform showed that 51.7% of adolescents posted a seductive picture, and that 20.4% posted an image in revealing clothing (when stratified by gender 39.2% of girls had posted an image in revealing clothing as opposed to 1.3% of boys) [10]. Butkowski, Dixon, Weeks and Smith [4] found that young adult women commonly employ a variety of stereotypical gender displays in their Instagram selfies. Poses that were more frequently observed in the sexualized self-presentation of young adult women were canting (e.g., as bending of the head/body as a sign of appeasement) and loss of control (e.g., as emotional expression of psychological disengagement). These self-sexualized images were more likely to receive likes and additional followers [4].

Adolescents are frequently exposed to sexualized messages and images, and are very sensitive about their appearance, because of their developmental stage during which peer and romantic relationships become increasingly important [11]. Therefore, they may also be more likely to engage in the posting of sexualized material. One longitudinal study found that adolescent girls who engaged in sexualized self-presentation on social networking sites had a higher willingness to engage in sexting [12]. A potential explanation for this finding is the fact that engaging in sexy self-presentation may increase adolescents’ self-confidence and could in turn increase their willingness to engage in sexting [12]. Perhaps sexy self-presentation on social media platforms could act for some adolescents as a way to experiment online with their sexuality and to engage in a form of “pre-sexting” [13]. Some adolescents may perceive negative consequences when engaging in sexual self-presentation on social media, especially girls, who can often fall victim to sexualized harassment and bullying (i.e., so-called “slut-shaming”), which are often similar to the consequences that victims of non-consensual sexting face [14,15,16].

## 3. The Prototype Willingness Model

The main idea of the PWM framework is that engagement in risk behaviors is not necessarily intentional or planned, as is posited by other theoretical approaches, such as the theory of planned behavior [17]. Therefore, the PWM also considers a social reaction path, which is more intuitive, in addition to a rational path [8]. Within the reasoned path, the intention to engage in the behavior is based on an analysis of one’s own attitudes towards the behavior and the perceived social norms. With regard to one’s own attitudes, individuals will evaluate whether they hold favorable or unfavorable attitudes towards a certain phenomenon. The subjective norms consist of the perceptions of the attitudes that important people in the life of adolescents hold [6]. The opinions of peers become increasingly important during adolescence [18]. Prior literature found that online risk behaviors are influenced by peer norms. Examples of these include, amongst others, online self-disclosure on social networking sites [6], online sexual risk behaviors [19], or sexting behavior [20,21]. Adolescents may perceive that the posting of sexually explicit images of themselves could lead to higher peer popularity [22].

In addition to peers, parents can also be an important source of social norms for adolescents. Some parents may restrict their children from posting sexually explicit images on social media, while other parents may have less restrictive attitudes towards their children’s social media use [6,23]. Therefore, the perceived parental norms are an additional influence on adolescents’ behaviors. As a last component of the reasoned path, the PWM posits that individuals who hold a positive intention to engage in a behavior also have a higher chance to engage in the behavior [17,20]. We propose the following hypotheses:

**H1:**  *The more positive the adolescents’ attitudes toward sexy online self-presentation, the greater their intention to post such images.*

**H2:**  *The more favorable the subjective norm of peers (H2a) and parents (H2b) towards posting sexy images, the higher the intention of adolescents to post such images on Instagram.*

**H3:**  *The higher the adolescents’ intention to post sexy images on Instagram, the higher their participation in the behavior.*

The PWM also comprises of a social reaction pathway, as decisions can be spontaneous and not always deliberately planned [8]. Although adolescents may not always intend to engage in a certain behavior, they may be willing to do so under specific situations. The willingness, if the occasion arises, to engage in a behavior can predict engagement in the behavior independently from their intention to do so [20,24]. The latter is especially the case when it comes to risky behaviors, such as posting sexy images on social media. Additionally, the two constructs intention and willingness are interconnected. We therefore hypothesize the following:

**H4:**  *The higher the adolescents’ willingness to engage in the posting of sexy images on Instagram, the higher their intention to engage in the behavior (H4a) and their actual participation in the behavior (H4b).*

The PWM further posits that adolescents’ engagement in risk behavior, such as the posting of sexualized images, can be impacted by how they perceive and identify with their prototypical peers engaging in this behavior (i.e., how they perceive the prototypical image of the average peer that posts sexy images on Instagram). When they engage in this behavior, they are aware that they could obtain the image that individuals who engage in this behavior hold [8]. Therefore, the “image” that adolescents hold of the typical peer who engages in this behavior, and the extent they identify with this person, may also influence their willingness to post sexy images themselves. Based on these theoretical assumptions, we hypothesize the following:

**H5:**  *The more favorable the adolescents perceive the prototype of someone posting sexy images on Instagram, the higher their willingness to do so themselves.*

**H6:**  *The more adolescents identify themselves with the prototype of peers posting sexy images on Instagram, the higher their willingness to do so themselves.*

Finally, according to the PWM, one’s own attitudes and the perceived positive social norms will not only affect individuals’ intention to engage in the behavior, but will also be more positively associated with the willingness to engage in a behavior, such as the posting of sexualized selfies. We therefore also add the following hypotheses:

**H7:**  *The more positive the adolescents’ attitudes toward sexy online self-presentation, the higher their willingness to post such images.*

**Figure 1:**
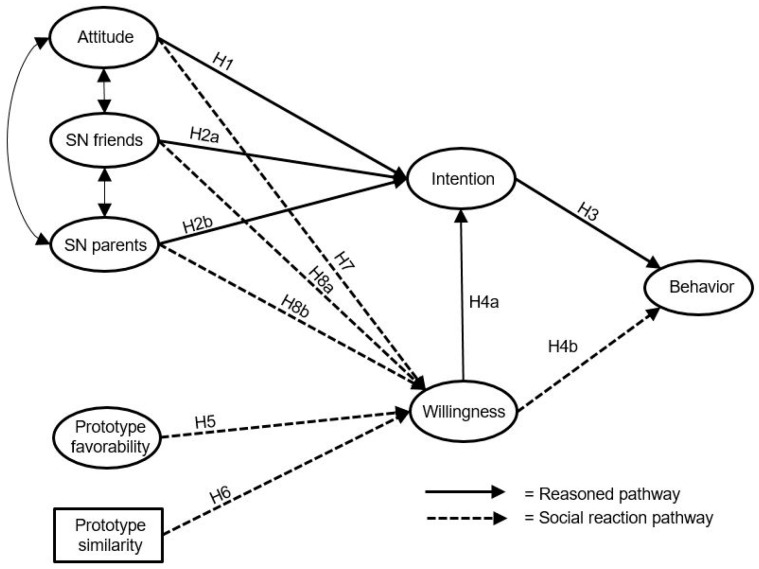
Conceptual model.

**H8:**  *The more favorable the subjective norm of peers (H8a) and parents (H8b) towards posting sexy images, the higher the willingness of adolescents to post such images on Instagram (see Figure 1).*

In sum, the posting of self-sexualized images on social media websites, such as Instagram, can be considered to be a risky behavior, as it may lead to subsequent bullying and harassment [14]. It may also be a form of pre-sexting that may lead to sexting behaviors afterwards [13]. Despite its potential consequences, more research from a theoretical perspective could provide additional insights into the behavior. The PWM will allow us to generate a deeper theoretical understanding of this phenomenon and will allow us to formulate recommendations for e-safety and media literacy education. Given that prior research has found gender differences in the way that boys and girls approach the posting of sexualized images and also perceive the consequences [12,14], we have stratified our analyses across gender.

## 4. Methods

### 4.1. Sample and Procedures

We conducted a paper-and-pencil survey as part of the Teen Online Relationship and Online Self-disclosure study. The study was conducted among 2626 students (*n* = 1530; 58.4% girls) between the ages of 14 and 21 (*M* = 16.14; *SD* = 1.02) in 10 secondary schools in the Dutch-speaking community in Belgium. The self-administered questionnaire was conducted during class time. The study was anonymous, and the students were told that their participation was voluntary and that their responses would remain anonymous and confidential. The participants were given envelopes to improve their privacy. The study’s procedures were approved by the ethical committee of the first author’s institution—the Ethical Committee for Social Sciences and Humanities at the University of Antwerp (SHW_15_23).

### 4.2. Measures

Following the recommendations by Ajzen and based on other studies applying the PWM [6,20], we developed a questionnaire to assess the constructs. In a short introduction, respondents were explained that the items concerned posting “sexy images of themselves on Instagram. We mean images that fit within one or more of the following categories: with a sexy gaze/with a sexy appearance/in a sexy posture”. The definition that was provided to participants is consistent with the conceptualization by Van Oosten et al. [9]. All items are presented in Table 1.

#### 4.2.1. Attitudes

Attitudes were measured by asking “For me, posting a sexy image of myself on Instagram is…” using four semantic differential items ranging on a five-point scale: (1) “bad-good”, (2) “disadvantageous-advantageous”, (3) “harmful-harmless”, (4) “a bad idea-a good idea”. The scale was reliable (*α* = 0.88).

#### 4.2.2. Subjective Norm

The subjective norm of friends and parents was measured with two items each: “I think my [friends/parents] would approve if posted sexy images of myself on Instagram” and “I think that generally speaking my friends/parents would not mind if I posted sexy images of myself on Instagram”. The four items were measured using a six-point Likert scale with item responses ranging from 1 = *strongly disagree* to 6 = *strongly agree*. Both scales were reliable, with *α* = 0.83 for friends/classmates, and *α* = 0.90 for parents.

#### 4.2.3. Intention

We measured the intention to post sexy images on Instagram using three items: “I am inclined to post sexy images of myself on Instagram”, “There is a chance that I would post sexy images of myself on Instagram”, “I want to share sexy images of myself on Instagram”, scored on a six-point Likert scale ranging from 1 = *strongly disagree* to 6 = *strongly agree*. The reliability of the scale was good (*α* = 0.89).

#### 4.2.4. Prototype Favorability

We presented the respondents with a short introduction that provided a description of a prototype: “Many youth post sexy images of themselves on Instagram. I want to know what you think about them. I am not suggesting anyone in particular, just someone your age who posts or would post sexy images of themselves on Instagram. Can you indicate to which extent you believe that the following characteristics fit them?” They were asked to rate the favorability of the image using the following three adjectives: “Popular”, “Sexy”, “Adventurous”, each followed a seven-point Likert scale ranging from 1 = *not at all* to 7 = *totally*. The resulting construct was reliable (*α* = 0.69).

#### 4.2.5. Prototype Similarity

The construct of prototype similarity was assessed using a single item: “How similar do you think you are to someone who posts sexy images of themselves on Instagram?” scored on a six-point scale, ranging from 1 = *not at all* to 6 = *totally*.

#### 4.2.6. Willingness

We presented the respondents with the description of a hypothetical scenario to measure the concept of willingness: “Imagine that you would be looking at your Instagram feed and you would notice that many of your friends are posting sexy images of themselves. What would you do?” The respondents were then asked the extent to which they would be engage in four hypothetical situations in this situation (e.g., “I would react and also post sexy images of myself on Instagram”). They were asked to score these scenarios on a six-point Likert scale ranging from 1 = *totally disagree* to 5 = *totally agree.* The internal consistency was good (*α* = 0.95).

#### 4.2.7. Behavior

Behavior was measured using three items from the construct “Sexy online self-presentation”, which was developed by van Oosten and Vandenbosch [12], asking respondents how often in the past 6 months they had posted an image on Instagram in which they were portrayed as the following: “with a sexy gaze”, “with a sexy appearance” and “in a sexy posture”. The items were measured on a seven-point Likert scale ranging from 1 = *never* to 7 = *always*. The internal consistency was good (*α* = 0.85).

### 4.3. Data Analytic Strategy

Structural equation modelling (SEM) was applied to the collected data using Mplus 8 to examine the relationships among the concepts of our model [25]. The analyses were performed using the following approach. First, a measurement model was built to test whether the observed variables reliably reflect the hypothesized latent variables. Thereafter, we estimated a structural model. Structural equation modelling results were obtained with maximum likelihood mean adjusted because preliminary tests suggested that self-reported stimulant behavior was a not normally distributed dependent variable.

The model fits of the measurement and path models were evaluated according to several fit indices. Given that the χ^2^ is almost always significant and not an adequate test of the model fit [26], we also report the comparative fit index (CFI) [27], Tucker–Lewis index (TLI) [28], root mean square error of approximation (RMSEA) [29] and the standardized root mean square residual (SRMR, [26]). The CFI and TLI range from 0 to 1.00, with a cut-off of 0.95 or higher indicating that the model provides a good fit and 0.90 indicating that the model provides an adequate fit [30,31]. RMSEA values below 0.05 indicate a good model fit, and values between 0.06 and 0.08 indicate an adequate fit [32]. The SRMR is a standardized summary of the average covariance residuals [26]. A good model fit is indicated when the SRMR is smaller than 0.08, and acceptable with values between 0.08 and 0.10 [31].

## 5. Results

The descriptions of the variables are presented in Table 1. Table 2 displays the correlations between the research constructs used in the model. All constructs were significantly related to each other at the *p* < 0.001 level.

### 5.1. Measurement Model

The measurement model provided a good fit for the data χ^2^ (182) = 397.30, *p* < 0.001; CFI = 0.988, TLI = 0.984, RMSEA = 0.026 (CI: 0.023–0.030), SRMR = 0.020. All variables were treated as latent constructs, with exception of the single-item measure prototype similarity. All factor loadings were significant and above 0.55. We subsequently included the age and gender as covariates in the analyses and examined the relationships between the age and gender of the adolescents and the study variables. If they were significantly related, they were added to the structural model. Age was associated with behavior and prototype similarity. Gender was used as a multigroup variable.

**Figure 2:**
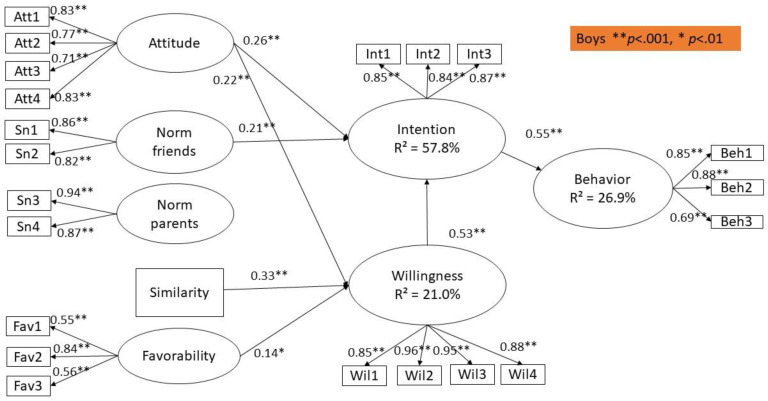
Structural model for boys. ** *p* < 0.001, * *p* < 0.01.

**Figure 3:**
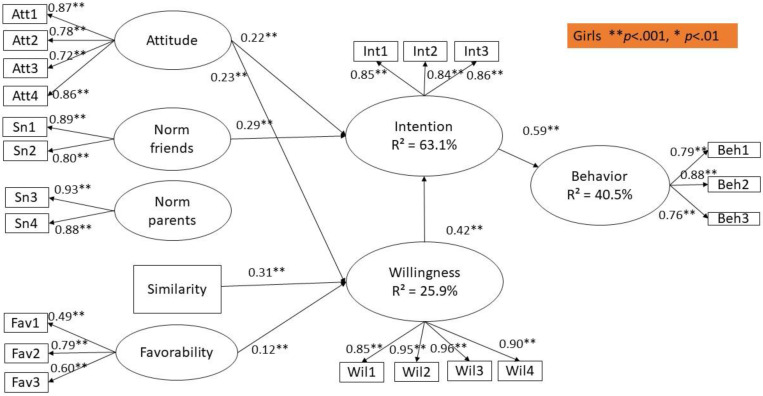
Structural model for girls. ** *p* < 0.001, * *p* < 0.01.

The age of the respondents was only significantly associated with adolescent’s posting of sexy pictures on Instagram (*β* = 0.09, *p* < 0.001) and prototype similarity (*β* = 0.07, *p* = 0.003). The gender of the adolescents was significantly related to behavior (*β* = −0.08, *p* = 0.002), attitudes (*β* = −0.22, *p* < 0.001), subjective norm of the friends (*β* = −0.15, *p* < 0.001), subjective norm of the parents (*β* = −0.27, *p* < 0.001), prototype favorability (*β* = −0.30, *p* < 0.001) and prototype similarity (*β* = −0.11, *p* < 0.001). Given the significant association between gender and all constructs, we decided to conduct a multi-group structural model with gender as the grouping variable (see Figure 2 and Figure 3). For the purpose of clarity, we have presented the results in separate figures. Between the groups and for each path, one-by-one comparisons of the constrained model to the unconstrained model were made to test whether the strength of the pathways differs among boys and girls. Chi-square difference tests revealed no significant differences between both groups.

### 5.2. Structural Model

The results of the structural model are presented in Figure 2. The results of the fit statistics indicate a sufficient model fit: χ^2^ (452) = 1135.25, *p* < 0.001; CFI = 0.965, TLI = 0.960, RMSEA = 0.042 (CI: 0.039–0.045) and SRMR = 0.090.

Our analyses reveal that attitude, subjective norms and willingness explain 57.8% and 63.1% of the variance in intention for boys and girls, respectively. In addition, prototype favorability and similarity account for 21.0% and 25.9% of the variance in willingness for boys and girls, respectively. Intention, willingness and gender account for 26.9% and 40.5% of the variance in male and female adolescent’s posting of sexy pictures on Instagram.

### 5.3. Sexualized Self-Presentation on Instagram for Boys

As shown in Figure 2, adolescent boys’ posting behavior on Instagram is significantly predicted by intention (*β* = 0.55, *p* < 0.001), but not by willingness (*β* = −0.07, *p* = 0.29). Furthermore, the intention to post pictures on Instagram is mostly influenced by willingness (*β* = 0.53, *p* < 0.001), along with attitude (*β* = 0.26, *p* < 0.001) and subjective norm of the friends (*β* = 0.21, *p* < 0.001). Thus, participants who have a more positive attitude towards posting pictures on Instagram discerned more social pressure from their friends, and those who are more willing to post pictures are likely to have a higher intention to post sexy pictures on Instagram. Contrary to our expectations, the subjective norm of the parents was not significantly associated with the intention of boys to post sexualized images of themselves (*β* = 0.04, *p* = 0.45). Furthermore, prototype similarity (*β* = 0.33, *p* < 0.001) is the strongest predictor of adolescent boys’ willingness to post sexy pictures on Instagram, followed by attitude and prototype favorability (*β* = 0.22, *p* < 0.001 and *β* = 0.14, *p* < 0.01, respectively). Thus, boys perceiving the prototype to be positive and resembling the self, and those who have a more favorable attitude towards posting on Instagram, are more willing to post sexy pictures on Instagram. Contrary to our expectations, subjective norm of the friends (*β* = −0.05, *p* = 0.56), and parents (*β* = 0.08 *p* = 0.29) were not significantly associated with boy’s willingness to post sexy pictures on Instagram. Age was not significantly associated with behavior (*β* = 0.07, *p* = 0.052), but it was associated with prototype similarity (*β* = 0.11, *p* < 0.01), meaning that older adolescents were more likely to perceive themselves as similar to youth who post sexy images of themselves on Instagram.

### 5.4. Sexualized Self-Presentation on Instagram for Girls

In this section, we discuss the PWM for girls, which is presented in Figure 3. Similar to the model for boys, adolescent girls’ posting behavior on Instagram is significantly predicted by intention (*β* = 0.59, *p* < 0.001), but not by willingness (*β* = 0.06, *p* = 0.12). Furthermore, the intention to post sexualized images on Instagram is mostly influenced by willingness (*β* = 0.42, *p* < 0.001), along with attitude (*β* = 0.22, *p* < 0.001) and the subjective norm of the friends (*β* = 0.29, *p* < 0.001). Similar to the model of boys, girls who felt social pressure from their friends also had a higher intention to post sexualized images of themselves on Instagram. The subjective norm of adolescent girls’ parents was also not significantly associated with their intention to post sexy images on the platform (*β* = 0.09, *p* = 0.051). Furthermore, prototype similarity (*β* = 0.31, *p* < 0.001) is the strongest predictor of adolescent girls’ willingness to post sexy pictures on Instagram, followed by attitude and prototype favorability (*β* = 0.22, *p* < 0.001 and *β* = 0.12, *p* < 0.001, respectively). Thus, girls perceiving the prototype to be positive and resembling the self, and those who have a more favorable attitude towards posting on Instagram, are more willing to post sexy pictures on Instagram. Contrary to our expectations, and similarly to the model for boys, the subjective norm of the friends (*β* = 0.07, *p* = 0.20), and parents (*β* = 0.05 *p* = 0.344) were not significantly associated with girls’ willingness to post self-sexualized images on Instagram. Being older was significantly associated with posting sexualized images on Instagram (*β* = 0.09, *p* = 0.000), but it was not associated with prototype similarity (*β* = 0.05, *p* = 0.080), meaning that older adolescent girls were not more likely to perceive themselves as a typical person who posts sexy images on Instagram.

## 6. Discussion

This study investigated the posting of sexualized images on social media using the prototype willingness model [8]. As the posting of sexualized images on social media can lead to sexualized bullying and harassment [14], the aim of our study was to generate a better understanding of the contextual antecedents of this behavior.

We found that older adolescents were more likely to post sexy images of themselves on Instagram. We also found that girls were significantly more likely to post self-sexualized images of themselves than boys. There is significant socio-cultural pressure on girls and young adult women to represent themselves in a sexualized way, especially from the media and consumer culture, yet society significantly penalizes them once they do so [33,34,35]. These beliefs, known as sexual double standards, are the basis for the practice of women’s shame, which involves denigrating women for a supposedly sexual act or insinuation [36]. Further studies could investigate, if similar to sexting, some girls may post sexy images of themselves on social media because they feel it is expected of them [37].

Nevertheless, the models for why boys and girls post sexual images of themselves on Instagram were very similar. The attitudes of peers were an important influential factor on adolescents’ willingness and intention to post sexualized selfies on Instagram, so hypotheses one (the more positive the adolescents’ attitudes toward sexy online self-presentation, the greater their intention to post such images) and seven (the more positive the adolescents’ attitudes toward sexy online self-presentation, the higher their willingness to post such images) were confirmed. This reinforces previous studies that found that adolescents’ intention to engage in this type of sexual behavior is associated with positive attitudes towards this practice [20,21,38]. It is unclear to what extent adolescents’ attitudes may be influenced by their media consumption. Prior research has found that adolescents’ sexting behavior could be influenced in part by their media consumption [39,40]. In fact, research on self-sexualization also found that adolescents’ behavior is influenced by media consumption (e.g., magazine reading among girls) [41]. However, our study was limited to the concepts of the PWM, future work could also apply a variety of theoretical frameworks, such as the social learning theory [42] or the media practice model [43] to investigate the antecedents of the sharing of sexualized images on social media. The media practice model could focus on the effects of mass-media consumption, as this has been found to be a contributing factor to adolescents’ self-sexualization behaviors [9].

The subjective peer norm was also shown to be significantly related to the intention of adolescents to post such images on Instagram, but not to the willingness to engage in that behavior. The subjective parental norm, on the other hand, was not significantly related to the intention or willingness to post sexualized images on that social network. Therefore, hypothesis two (the more favorable the subjective norm of peers and parents towards posting sexy images, the higher the intention of adolescents to post such images on Instagram) is half confirmed and hypothesis eight (the more favorable the subjective norm of peers and parents towards posting sexy images, the higher the willingness of adolescents to post such images on Instagram) are rejected. The exchange and display of sexual content is increasingly normalized among adolescents and young people [39,44,45], so the perception of this behavior as a widespread peer standard may facilitate the intention to engage in this behavior. This is in line with research on sexting, in which it was also found that the perceived opinions of peers play an important role in adolescents’ sexting behavior [20,21,46,47]. The subjective norm is one of the strongest predictors of young people’s engagement in digital sexual behaviors [20,21,38], so peer norms play a crucial role in the publication by adolescents of sexual photos of themselves and others on the internet [48]. Therefore, the results of the study confirm the importance of including the subjective peer norm in education programs, just as educational plans such as “Asegúrate” are doing [49]. It is essential, then, to encourage reflection on social pressure, the need for popularity, and a critical attitude towards this type of sexual behavior through the internet [50]. It would also be advisable to start from the perceptions and experiences of adolescents in a way that facilitates the understanding of this practice and the motivations behind it in order to be able to respond to the real needs of young people [51].

Remarkably, parents’ opinions on the posting of sexualized images does not affect their willingness or intention to post sexualized selfies. One of the potential explanations may be that at the time of our study, most parents were not active on Instagram and adolescents may not allow their parents access to their profile on Instagram [6]. Another potential explanation may be that parents do not communicate with their children about their posting behavior on social media, and perhaps especially, the posting of self-sexualizing images. Given that the parental norms may be unspoken, they may also have a limited impact on adolescents’ behavior [23]. One strategy to increase the influence of parents is maybe to increase their skills in communicating about these issues. It is important that schools conduct individual counselling and meetings with families to advise and train them about the internet and children’s use of it and social networks [52], so that they feel competent in this type of action [53]. Families should talk about these issues with their children, as the family context can have an impact on adolescents’ safer internet use [23,54].

The willingness to post sexy images on Instagram was shown to be significantly related to the intention to do this behavior and the latter, in turn, to their involvement in the behavior, so we confirmed hypothesis three (the higher the adolescents’ intention to post sexy images on Instagram, the higher their participation in the behavior) and hypothesis four (the higher the adolescents’ willingness to engage in the posting of sexy images on Instagram, the higher their intention to engage in the behavior and their actual participation in the behavior). Meta-analyses have often found high correlations between intention and behavior [55] and this study also confirms that willingness helps to understand and predict the occurrence of behavior. When there is an opportunity to engage in a risky behavior, social image influences adolescents’ decisions [56].

The prototype favorability and similarity were important influential factors on adolescents’ willingness to post sexualized selfies on Instagram, so hypotheses five (the more favorable the adolescents perceive the prototype of someone posting sexy images on Instagram, the higher their willingness to do so themselves) and six (the more adolescents identify themselves with the prototype of peers posting sexy images on Instagram, the higher their willingness to do so themselves) were confirmed. These results coincide with previous studies that have found significant relationships between prototypes and the willingness to behave [57,58]. At this point, the social cognitive theory [59,60] can help explain this influence. This theory holds that people guide their behavior based on the behaviors they observe in others, so they may have adopted certain behaviors because they observed them in others. In addition, they can identify and reinforce their own values and are more likely to replicate those behaviors when they are socially reinforced, for example, through "likes" and comments on social networking sites [61]. Gaining popularity and acceptance from peers is very important for adolescents. This underscores the importance of taking into account the peer context when promoting a critical and reflective attitude towards the models found on the internet [50,62]. Future research should investigate the effects of Instagram use on body image. Since Instagram is replete with content that often represents very thin bodies that supposedly represent ideal body standards [63], adolescents may tend to try to obtain said bodies, creating psychological or physical difficulties in the short or long term, so this risk must also be addressed through education.

Some limitations of our study should be kept in mind when interpreting the results of our study. First, the study used a cross-sectional design. Future research could employ a longitudinal design that would allow us to capture and study changes in behavior over time. Second, our study investigated the behaviors of older secondary school students, between the ages of 14 and 21 years old. Future work could also investigate the posting behaviors of younger adolescents, especially as they may be more vulnerable for the risks involved with posting these images on Instagram. Particularly as research on sexting found that young teenagers are sometimes involved in a type of “pre-sexting”, during which they experiment with taking sexy images of themselves, and therefore future research could include younger adolescents [13]. Third, our study focused on the posting of sexualized images on Instagram, future work could compare different platforms and evaluate if there are also differences in behaviors between different types of platforms, which may either be focused on ephemeral content (such as Snapchat) or that are more focused on videos (such as TikTok). Fourth, as with all survey research, some respondents may have provided socially desirable answers. Future studies on the influence of Instagram on the posting of sexualized images could use alternative types of data collection (e.g., observational data). A final limitation of our study is that it was conducted in Belgium, future research could also apply a cross-cultural or cross-national approach, as other nations and cultures may have different attitudes and social norms towards the display of sexuality on social media.

## 7. Conclusions

Posting sexy images on social media can be considered as a risk behavior for adolescents. It can have long term consequences and could be a first step towards sexting behavior. In this study, we investigate the associations between sexualized self-presentation on Instagram and the concepts of the prototype willingness model. The results show that older adolescents and girls were more likely to post images of themselves on Instagram. The models showed that peer norms and willingness and attitudes were significantly associated with posting intention. The perceived norms of adolescents’ parents were not significantly related to the behaviors. There were also significant associations between perceived similarity, prototype favorability and the willingness to engage in sexualized self-presentation on Instagram.

These results underscore the need for education campaigns to discuss the potential risks of engaging in sexualized self-presentation on social media. As older adolescents are more inclined to post sexy pictures of themselves online and as they will enter higher education and/or the job market, they could be educated about the potential impact of their pictures when they start higher education or apply for a job or internship [53]. Increasingly, human resources managers are engaging in cybervetting—the screening of social media in search for information about job applicants in order to evaluate their suitability for a specific position [64,65]. It is therefore important that young people are informed about the impression they could leave on potential employers when they perform online searches concerning potential job or internship applicants. This can be complicated to convey to teenagers, because it may be outside their circle of interests and concerns. However, given the age at which they begin to publish sexual content, it is critical to address it in an educational manner early on. For educators, this will also reflect a difficult balance between encouraging creativity and self-expression and making adolescents aware of the broader perceptions of certain types of content.

Programs should be constructive and based on children’s ideas [66]. In fact, adolescents consider that messages from their environment and the media influence their willingness to participate in this type of phenomenon by implying that it is a normal practice [67], so it is important that future education strategies address media analysis from a critical perspective [68], while acknowledging the importance of being able to express one’s sexuality without the judgement of others.

## Figures and Tables

**Table 1:** Descriptions of the study variables.

	*M*	*SD*
*Attitudes*		
Att1. Bad-good	2.13	0.94
Att2. Disadvantageous-advantageous	2.15	0.94
Att3. Harmful-harmless	2.32	1.07
Att4. A bad idea-a good idea	2.00	0.94
*Subjective norm-Friends*		
Sn1. I think my friends would approve if posted sexy images of myself on Instagram	2.89	1.38
Sn2. I think that generally speaking my friends would not mind if I posted sexy images of myself on Instagram	3.32	1.39
*Subjective norm-Parents*		
Sn3. I think my parents would approve if posted sexy images of myself on Instagram	2.01	1.19
Sn4. I think that generally speaking my parents would not mind if I posted sexy images of myself on Instagram	2.08	1.26
*Prototype favorability*		
Fav1. Popular	4.44	1.55
Fav2. Sexy	4.08	1.56
Fav3. Adventurous	3.34	1.56
*Prototype similarity*		
How similar do you think you are to somebody who posts sexy images of themselves on Instagram “not at all-totally”	1.93	1.04
*Willingness*		
Wil1. After some hesitation, I would also post sexy images of myself on Instagram	1.79	1.00
Wil2. I would do the same and post sexy images of myself on Instagram	1.64	0.91
Wil3. I would also post sexy images of myself on Instagram	1.65	0.94
Wil4. I would react and also post sexy images of myself on Instagram	1.65	0.95
*Intention*		
Int1. I am inclined to post sexy images of myself on Instagram	1.72	0.97
Int2. There is a chance that I would post sexy images of myself on Instagram	2.17	1.23
Int3. I want to share sexy images of myself on Instagram	1.72	0.99
*Behavior* How often in the past 6 months have you posted images of yourself in which you were depicted with…		
Beh1. With a sexy gaze	1.52	1.01
Beh2. With a sexy appearance	1.60	1.11
Beh3. In a sexy posture	1.30	0.83

**Table 2:** Correlations between the research constructs.

	1	2	3	4	5	6	7	8
Attitude	/							
Social norm friends	0.67 **	/						
Social norm parents	0.61 **	0.73 **	/					
Intention	0.61 **	0.60 **	0.50 **	/				
Willingness	0.42 **	0.36 **	0.32 **	0.67 **	/			
Prototype favorability	0.37 **	0.36 **	0.29 **	0.36 **	0.31 **	/		
Behavior	0.46 **	0.36 **	0.33 **	0.56 **	0.38 **	0.18 **	/	
Prototype similarity	0.39 **	0.36 **	0.33 **	0.53 **	0.44 **	0.34 *	0.39 **	/

** *p* < 0.001, * *p* < 0.01.
